# Socio-economic differences in body mass index: the contribution of genetic factors

**DOI:** 10.1038/s41366-024-01459-w

**Published:** 2024-01-10

**Authors:** Karri Silventoinen, Hannu Lahtinen, Fanny Kilpi, Tim T. Morris, George Davey Smith, Pekka Martikainen

**Affiliations:** 1 University of Helsinki, Faculty of Social Sciences, Population Research Unit, Helsinki, Finland; 2 Max Planck – University of Helsinki Center for Social Inequalities in Population Health, Helsinki, Finland; 3 Bristol Medical School, University of Bristol, Population Health Sciences, Bristol, UK; 4 MRC Integrative Epidemiology Unit, University of Bristol, Bristol, UK; 5 Centre for Longitudinal Studies, Social Research Institute, University College London, London, UK; 6 Max-Planck-Institute for Demographic Research, Rostock, Germany

**Keywords:** Genetics, Risk factors

## Abstract

**Background:**

Higher mean body mass index (BMI) among lower socioeconomic position (SEP) groups is well established in Western societies, but the influence of genetic factors on these differences is not well characterized.

**Methods:**

We analyzed these associations using Finnish health surveys conducted between 1992 and 2017 (*N* = 33 523; 53% women) with information on measured weight and height, polygenic risk scores of BMI (PGS-BMI) and linked data from administrative registers to measure educational attainment, occupation-based social class and personal income.

**Results:**

In linear regressions, largest adjusted BMI differences were found between basic and tertiary educated men (1.4 kg/m^2^, 95% confidence interval [CI] 1.2; 1.6) and women (2.5 kg/m^2^, 95% CI 2.3; 2.8), and inverse BMI gradients were also found for social class and income. These SEP differences arose partly because mean PGS-BMI was higher and partly because PGS-BMI predicted BMI more strongly in lower SEP groups. The inverse SEP gradients of BMI were steeper in women than in men, but sex differences were not found in the genetic contributions to these differences.

**Conclusions:**

Better understanding of the interplay between genes and environment provides insight into the mechanisms explaining SEP differences in BMI.

## Introduction

The inverse association between socio-economic position (SEP) and body mass index (BMI) has been convincingly established in Western societies [1]. BMI is also influenced by genetic factors as shown by large scale twin [2] and genome-wide-association studies (GWAS) [3]. Genetic factors can influence SEP differences in BMI through two mechanisms. First, the same genetic variants may affect both BMI and SEP. The expression of many SNPs associated with BMI is enriched in the brain, especially in the hypothalamus, pituitary gland, hippocampus and limbic system [3]. These brain areas have an important role in appetite regulation, emotions, learning, cognition and memory, potentially affecting both BMI and SEP [4]. Second, SEP may modify the effect of genetic factors on BMI. Twin studies have shown that low parental education [5], own education [6] and income [7] are associated with the higher variation in BMI attributable to genetic factors. There is also evidence that polygenic scores for BMI (PGS-BMI) interact with SEP [8, 9], though not all studies have replicated this result [10].

A limitation of previous studies is that they have not analyzed simultaneously how these different genetic mechanisms contribute to SEP differences in BMI. Further, they have typically used only a single SEP indicator. In this study, we use a large population-based cohort to analyze (i) whether there are SEP differences in BMI using three SEP indicators formed during the life course and, if this is the case, (ii) to analyze if this is due to differences in mean PGS-BMI between the SEP categories or moderation of PGS-BMI measured BMI associations by SEP.

## Data and methods

Finnish population-based health surveys (FINRISK 1992, 1997, 2002, 2007 and 2012 surveys and Health 2000 and 2011 and FinHealth 2017 surveys) having response rates between 65% and 93% were pooled together [11]. BMI (kg/m^2^) was calculated from height and weight measured at the baseline health examination when the participants also gave DNA samples. These data were linked to population registers to assess three measures of SEP [12]: (i) the highest completed educational degree up to the end of 2019, (ii) occupational based social class at the age of 40, or if missing, the most recent previous measurement when the individual was employed, and (iii) income quintiles based on the mean of yearly percentiles of personal taxable income at 35–40 years of age.

We restricted our sample to those born between 1935 and 1980 due to the availability of SEP indicators, and those between 25 and 70 years at the time of the survey because of age-related declines in BMI after age 70 [13]. After randomly removing one individual from pairs with identity-by-descent proportion ≥0.178 (corresponding to the expected lower bound of second-degree relatives; *N* = 1844), we had 33,523 participants (53% women) in our study sample. We removed 3790 participants from social class models and 4035 participants from income models due to missing information. PGS-BMI was derived from the GWAS by Yengo et al. [14]. SBayesR was used to adjust for linkage disequilibrium [15]. In our cohort, PGS-BMI explained 14% of BMI variance in men and 15% in women. The data were analyzed by linear regression models using Huber-White standard errors to adjust for the potential heteroscedasticity of residuals adjusting for age, age square, region of residence, 10 first principal components of population structure, and survey round—genotyping batch combination. Supplementary table 1 presents descriptive statistics of the variables. Linkage disequilibrium adjustment of PGS-BMI GWAS scores was conducted with GCTB 2.03; genetic principal components, genetic relatedness and PGS-BMI with PLINK 1.9–2.0; and all statistical models with Stata, version 16.1.

## Results

Table 1 presents the model predicted means of BMI by SEP indicators. BMI showed clear gradients over all SEP indicators in men and women (*p* < 0.00001), where more advantaged SEP was associated with lower BMI. The largest BMI difference was found between basic and higher tertiary educated men (1.4 kg/m^2^, 95% confidence interval [CI] 1.2; 1.6) and women (2.5 kg/m^2^, 95% CI 2.3; 2.8). The BMI gradients were larger in women than in men for all SEP indicators (*p*-values of sex-interactions <0.00001).

**Table 1:** Model predicted BMI by socioeconomic position indicators and sex^a^.

	Men			Women		
	Mean	95% confidence intervals		Mean	95% confidence intervals	
		LL	UL		LL	UL
Education						
Basic	27.6	27.5	27.8	27.5	27.3	27.7
Secondary	27.3	27.2	27.4	26.7	26.6	26.8
Lower tertiary	27.0	26.9	27.2	26.0	25.8	26.1
Higher tertiary	26.2	26.1	26.4	25.0	24.8	25.1
*p*-value: main effect^b^	<0.00001			<0.00001		
*p*-value: sex-interaction^c^	<0.00001					
Social class						
Manual	27.3	27.1	27.4	26.9	26.8	27.1
Lower non-manual	27.0	26.8	27.1	26.1	26.0	26.2
Upper non-manual	26.6	26.5	26.8	25.3	25.1	25.5
Self-employed	27.6	27.3	27.8	27.0	26.7	27.2
Farmers	27.8	27.3	28.4	27.2	26.3	28.1
*p*-value: main effect^b^	<0.00001			<0.00001		
*p*-value: sex-interaction^c^	<0.00001					
Income						
Lowest quintile	27.3	27.0	27.5	26.8	26.6	27.0
4.quintile	27.3	27.1	27.5	26.4	26.3	26.6
3.quintile	27.2	27.1	27.4	26.1	26.0	26.3
2.quintile	27.1	27.0	27.2	25.7	25.5	25.8
Highest quintile	26.9	26.8	27.0	25.6	25.4	25.8
*p*-value: main effect^b^	<0.00001			<0.00001		
*p*-value: sex-interaction^c^	<0.00001					

*LL* lower limit, *UL* upper limit, *SEP* socioeconomic position.

^a^Predicted values based on linear models holding control variables (age, age squared, 10 first principal components of population structure, region of residence, and the combination of data collection and genotyping batch) at their observed values.

^b^*P-*values based on F-tests of the joint effect of the SEP categories.

^c^*P*-values based on F-tests of the joint effect of the SEP*sex interaction terms from sex pooled models.

In Panel A of Table 2, there were gradients in BMI predicted by PGS for all SEP indicators (*p* < 0.00001). The largest differences were found for education: the difference in BMI predicted by the PGS between basic and tertiary education was 0.57 (95% CI 0.48; 0.66) kg/m^2^ in men and 0.72 (95% CI 0.61; 0.84) kg/m^2^ in women. These differences were smaller than those found for BMI for all SEP indicators. Further, in contrast to BMI, the associations between BMI predicted by PGS and SEP indicators were roughly similar in men and women (*p*-values of sex-interactions ≥0.046).

**Table 2:** BMI predicted by PGS (Panel A) and the regression coefficients of BMI predicted by PGS on BMI (Panel B) by socioeconomic position indicators and sex^a^.

	Panel A: Mean BMI predicted by PGS						Panel B: Coefficients of BMI predicted by PGS on BMI					
	Men			Women			Men			Women		
	Mean	95% confidence intervals		Mean	95% confidence intervals		β	95% confidence intervals		β	95% confidence intervals	
		LL	UL		LL	UL		LL	UL		LL	UL
Education												
Basic	27.4	27.3	27.4	26.7	26.6	26.7	0.98	0.89	1.06	1.05	0.97	1.14
Secondary	27.3	27.2	27.3	26.5	26.5	26.6	1.07	1.00	1.13	1.02	0.96	1.08
Lower tertiary	27.1	27.1	27.2	26.3	26.3	26.4	0.94	0.86	1.02	0.94	0.87	1.00
Higher tertiary	26.8	26.7	26.9	25.9	25.9	26.0	0.85	0.75	0.95	0.75	0.66	0.83
*p*-value: main effect (Panel A)/SEP*PGS-interaction (Panel B)^b^	<0.00001			<0.00001			0.007			<0.00001		
*p*-value: sex-interaction^c^	0.131						0.179					
Social class												
Manual	27.4	27.3	27.4	26.6	26.6	26.7	1.01	0.94	1.08	1.04	0.96	1.12
Lower non-manual	27.1	27.1	27.2	26.4	26.4	26.5	1.03	0.94	1.11	0.97	0.92	1.03
Upper non-manual	27.0	26.9	27.0	26.1	26.0	26.2	0.89	0.81	0.97	0.83	0.76	0.91
Self-employed	27.2	27.1	27.3	26.5	26.4	26.6	1.05	0.92	1.19	1.10	0.97	1.24
Farmers	27.3	27.0	27.5	26.3	26.0	26.6	0.70	0.30	1.11	1.02	0.39	1.64
*p*-value: main effect (Panel A)/SEP*PGS-interaction (Panel B)^b^	<0.00001			<0.00001			0.113			0.0014		
*p*-value: sex-interaction^c^	0.046						0.352					
Income												
Lowest quintile	27.2	27.2	27.3	26.4	26.3	26.5	1.15	0.98	1.31	1.03	0.94	1.11
4.quintile	27.3	27.2	27.4	26.5	26.5	26.6	1.03	0.89	1.17	1.02	0.94	1.09
3.quintile	27.3	27.3	27.4	26.4	26.4	26.5	0.99	0.89	1.09	0.99	0.91	1.06
2.quintile	27.2	27.2	27.3	26.3	26.3	26.4	1.02	0.94	1.10	0.94	0.85	1.03
Highest quintile	27.1	27.1	27.2	26.2	26.1	26.3	0.90	0.84	0.96	0.83	0.72	0.94
*p*-value: main effect (Panel A)/SEP*PGS-interaction (Panel B)^b^	<0.00001			<0.00001			0.019			0.040		
*p*-value: sex-interaction^c^	0.161						0.788					

*LL* lower limit, *UL* upper limit, *SEP* socioeconomic position, *PGS* polygenic risk score.

^a^Predicted values based on linear models, adjusted by age, age squared, 10 first principal components of population structure, region of residence, and the combination of data collection and genotyping batch. BMI predicted by PGS is based on the prediction from sex-specific linear regression where BMI is regressed on PGS-BMI. Panel A reports marginal means holding control variables at their observed values. Panel B reports the coefficients of BMI predicted by PGS from SEP-stratified models.

^b^In panel A, *p*-values are based on F-tests of the joint effect of the SEP categories (“main effect”). In panel B, *p*-values are based on F-tests of the joint effect of the SEP*PGS-interaction terms from SEP pooled models (“SEP*PGS-interaction”).

^c^In panel A, *p*-values are based on F-tests of the joint effect of the SEP*sex interaction terms from sex pooled models. In panel B, *p*-values are based on F-tests of the joint effect of the SEP*PGS*sex- second order interaction terms from SEP and sex pooled models including all respective first-order interactions.

In Panel B of Table 2, we analyzed how BMI predicted by PGS was associated with BMI in different social strata. The associations were consistently lower in the higher SEP categories. For example, whilst a one unit increase of BMI predicted by PGS was associated with a higher BMI of 0.85 (95% CI 0.75; 0.95) kg/m^2^ among men and 0.75 (95% CI 0.66; 0.83) kg/m^2^ among women with higher tertiary education, the corresponding associations were 0.98 (95% CI 0.89; 1.06) kg/m^2^ among men and 1.05 (95% CI 0.97; 1.14) kg/m^2^ among women with basic education (the p-value of the gradient 0.007 for men and <0.00001 for women). The SEP gradients of the associations between BMI predicted by PGS and BMI were roughly similar in men and women (*p*-values of sex-interactions ≥0.179).

## Discussion

In this large population-based cohort study, we found that the SEP differences in BMI were partly explained by differences in PGS-BMI between the SEP categories. This is consistent with previous results that many genetic variants associated with BMI express in the brain areas important for cognition and memory [4], and that the genetic factors affect BMI largely through behavior, especially nutrition [16]. Further, cognitive function is associated with nutrition intake and obesity [17]. Since health behavior also contributes to the SEP differences in obesity [1], it is possible that genetic factors affect SEP differences in BMI because the same brain areas are associated with socioeconomic achievement and health behavior, especially nutrition but possibly also physical exercise, through cognitive function. The effect of same genetic factors on both BMI and SEP may continue across the life course as we found that BMI was associated with SEP indicators formed at different life stages: education typically in young adulthood and social class and income later in life. However, the genetic correlation may also emerge if BMI affects SEP through factors such as BMI related health conditions or body-size discrimination [18].

In addition to the differences in BMI predicted by PGS between SEP categories, we observed that PGS*SEP interaction effects existed whereby PGS-BMI was more strongly associated with BMI in lower than in higher SEP categories. Previous twin [5–7] and PGS studies [8, 9] have observed corresponding gene-environment interactions. However, uniquely, we demonstrated that both genetic correlations and interactions contributed to the SEP differences in BMI. There is previous evidence that both material and psychosocial stressors associated with lower SEP can lead to a higher risk of obesity [19]. Our results on the interaction between SEP and PGS-BMI support that these stressors may have a greater impact when there is a high genetic susceptibility to obesity. Although SEP gradients in BMI were stronger in women than in men, no sex difference was found in the genetic mechanisms behind the association between SEP and BMI. This observation suggests possible women-specific environmental factors which may stem from, e.g., higher pressure for weight control in women with high SEP because of higher BMI related discrimination in women as compared to men [1].

Strengths of this study include the large population-representative sample with a high response rate and possibility to use three SEP indicators formed at different phases of the life course. Further, since BMI was measured and SEP indicators were register-based, reporting bias should be minimized. However, the BMI-PGS used in this study accounted for only a part (~20%) of the total genetic BMI variation estimated using twin design [2]. Previous large-scale twin studies have suggested that obesogenic macro-environments can increase the variation of BMI attributable to genetic factors [2] and strengthen the interaction between SEP and genetic factors [5]. Thus, we expect that the results may be most similar in regions with a similar level of BMI (e.g., other European countries) whereas the associations can be stronger in regions with higher (e.g., the USA) and weaker in regions with lower level of BMI (e.g., Japan) [20]. Future comparative studies could address this hypothesis.

In conclusion, genetic factors appear to play a role behind SEP differences in BMI. These differences are partly due to the accumulation of genetic variants predisposing to high BMI in lower SEP categories, and partly because low SEP reinforces the effects of these genetic variants on BMI. Improving the understanding on the interplay between genes and environment can give insight into the mechanisms behind SEP differences in BMI.

### Supplementary information

Supplementary material

## Data Availability

The data underlying this article were provided by third party by permission and is not publicly available. Data will be shared on request to the corresponding author with permission of third party.

## Code availability

All computer codes are available from the corresponding author.

## References

[1] McLaren L (2007). Socioeconomic status and obesity. Epidemiol Rev.

[2] Silventoinen K, Jelenkovic A, Sund R, Yokoyama Y, Hur YM, Cozen W (2017). Differences in genetic and environmental variation in adult BMI by sex, age, time period, and region: an individual-based pooled analysis of 40 twin cohorts. Am J Clin Nutr.

[3] Turcot V, Lu Y, Highland HM, Schurmann C, Justice AE, Fine RS (2018). Protein-altering variants associated with body mass index implicate pathways that control energy intake and expenditure in obesity. Nat Genet.

[4] Lenard NR, Berthoud HR (2008). Central and peripheral regulation of food intake and physical activity: pathways and genes. Obesity.

[5] Silventoinen K, Jelenkovic A, Latvala A, Yokoyama Y, Sund R, Sugawara M (2019). Parental education and genetics of BMI from infancy to old age: a pooled analysis of 29 twin cohorts. Obesity.

[6] Johnson W, Kyvik KO, Skytthe A, Deary IJ, Sørensen TIA (2011). Education modifies genetic and environmental influences on BMI. PLoS One.

[7] Dinescu D, Horn EE, Duncan G, Turkheimer E (2016). Socioeconomic modifiers of genetic and environmental influences on body mass index in adult twins. Health Psychol.

[8] Tyrrell J, Wood AR, Ames RM, Yaghootkar H, Beaumont RN, Jones SE (2017). Gene-obesogenic environment interactions in the UK Biobank study. Int J Epidemiol.

[9] Hüls A, Wright MN, Bogl LH, Kaprio J, Lissner L, Molnár D (2021). Polygenic risk for obesity and its interaction with lifestyle and sociodemographic factors in European children and adolescents. Int J Obes.

[10] Bann D, Wright L, Hardy R, Williams DM, Davies NM (2022). Polygenic and socioeconomic risk for high body mass index: 69 years of follow-up across life. PLoS Genet.

[11] Paalanen L, Härkänen T, Tolonen H (2019). Protocol of a research project “Projections of the burden of disease and disability in Finland - health policy prospects” using cross-sectional health surveys and register-based follow-up. BMJ Open.

[12] Silventoinen K, Lahtinen H, Davey Smith G, Morris TT, Martikainen P. Height, social position and coronary heart disease incidence: the contribution of genetic and environmental factors. J Epidemiol Community Health. 2023;77:384–390. 10.1136/jech-2022-219907 36963814

[13] Calderón-Larrañaga A, Hu X, Guo J, Ferrucci L, Xu W, Vetrano DL (2021). Body mass trajectories and multimorbidity in old age: 12-year results from a population-based study. Clin Nutr.

[14] Yengo L, Sidorenko J, Kemper KE, Zheng Z, Wood AR, Weedon MN (2018). Meta-analysis of genome-wide association studies for height and body mass index in ~700000 individuals of European ancestry. Hum Mol Genet.

[15] Lloyd-Jones LR, Zeng J, Sidorenko J, Yengo L, Moser G, Kemper KE (2019). Improved polygenic prediction by Bayesian multiple regression on summary statistics. Nat Commun.

[16] Silventoinen K, Konttinen H (2020). Obesity and eating behavior from the perspective of twin and genetic research. Neurosci Biobehav Rev.

[17] Higgs S (2023). Is there a role for higher cognitive processes in the development of obesity in humans?. Philos Trans R Soc Lond B Biol Sci.

[18] Böckerman P, Cawley J, Viinikainen J, Lehtimäki T, Rovio S, Seppälä I (2019). The effect of weight on labor market outcomes: an application of genetic instrumental variables. Health Econ.

[19] Claassen MA, Klein O, Bratanova B, Claes N, Corneille O (2019). A systematic review of psychosocial explanations for the relationship between socioeconomic status and body mass index. Appetite.

[20] NCD Risk Factor Collaboration (NCD-RisC). Trends in adult body-mass index in 200 countries from 1975 to 2014: a pooled analysis of 1698 population-based measurement studies with 19·2 million participants. Lancet. 2016;387:1377–96.. 10.1016/S0140-6736(16)30054-X PMC7615134 27115820

