# Supplementary material for: Socio-economic differences in body mass index: the contribution of genetic factors
Source: Int J Obes (Lond). 2024 Jan 10;48(5):741–5. doi: 10.1038/s41366-024-01459-w (PMC11058309; doi:10.1038/s41366-024-01459-w)
Supplement: Supplementary file 1 — Supplementary material [file 41366_2024_1459_MOESM1_ESM.pdf]

Supplementary table 1. Proportion of participants and descriptive statistics of BMI by social position indicators and sex.

	Men			Women		
	%	BMI		%		
		Mean	SD		Mean	SD
**Education**						
Basic	25	27.9	4.4	20	28.0	5.5
Secondary	42	27.2	4.3	38	26.8	5.3
Lower tertiary	23	27.0	3.9	29	25.8	4.9
Higher tertiary	11	26.0	3.8	13	24.4	4.2
Total	100	27.2	4.2	100	26.4	5.2
**N**	15666			17857		
**Social class**						
Manual	43	27.3	4.3	24	27.2	5.4
Lower non-manual	21	26.9	4.0	48	26.1	5.0
Upper non-manual	21	26.5	3.7	19	24.9	4.5
Self-employed	13	27.6	4.5	8	27.3	5.5
Farmers	1	28.4	4.0	1	28.4	5.7
Total	100	27.1	4.2	100	26.2	5.1
N	13892			15841		
**Income**						
Lowest quintile	12	27.3	5.1	24	27.0	5.6
4.quintile	12	27.3	4.5	27	26.5	5.2
3.quintile	18	27.2	4.2	23	26.1	4.9
2.quintile	26	27.1	4.0	16	25.5	4.6
Highest quintile	33	26.9	3.8	9	25.3	4.5
Total	100	27.1	4.2	100	26.3	5.1
N	13774			15714		
